# Data in support of effects of cell–cell contact and oxygen tension on chondrogenic differentiation of stem cells

**DOI:** 10.1016/j.dib.2015.06.020

**Published:** 2015-07-08

**Authors:** Bin Cao, Zhenhua Li, Rong Peng, Jiandong Ding

**Affiliations:** State Key Laboratory of Molecular Engineering of Polymers, Department of Macromolecular Science, Advanced Materials Laboratory, Fudan University, Shanghai 200433, China

## Abstract

This paper presents data related to the research article entitled “Effects of cell–cell contact and oxygen tension on chondrogenic differentiation of stem cells” [1]. Three sets of micropatterns were fabricated to study the influence of the cell–cell contact on the chondrogenic induction of mesenchymal stem cells (MSCs). The basic repeat units of these micropatterns were of the same area and microisland number to guarantee the same cell density in each culture well. Cells on these micropatterns experienced the same microenvironment except cell–cell contact extent. Immunofluorescent staining and quantitative real-time polymerase chain reaction (qRT-PCR) were performed, and the data are included here.

**Specifications table**

**Table:** 

Subject area	Material sciences, chemistry, biology, regenerative medicine
More specific subject area	Biomaterials, micropattern, stem cell differentiation, chondrogenesis
Type of data	Table, figure
How data was acquired	Microscopy, PerlPrimer, Primer-BLAST
Data format	Raw for the figure and analyzed for the table
Experimental factors	Cell–cell contact, oxygen tension
Experimental features	The prepared micropatterns were captured by microscopy, and the sequence of primers were obtained by PerlPrimer and Primer-BLAST
Data source location	Fudan University, Shanghai, China
Data accessibility	Data is provided in the article

**Value of the data**

• Data can be helpful to the readers when designing a pattern with appropriate spatial distribution of microdomains of different microisland numbers to enhance efficiency to examine the effects of microisland numbers in microdomains on cell behaviors.
• Data emphasizes the importance of keeping the same microisland density in a basic repeated unit in cell studies in order to rule out the interference of other factors such as difference of paracrined soluble factors.
• The sequences of primers can be employed by other researchers in sequence design of targeted genes detected by quantitative real-time polymerase chain reaction (qRT-PCR) to study the chondrogenic induction of mesenchymal stem cells (MSCs) derived from bone marrow of Sprague Dawley (SD) rats.

## 1 Data, experimental design, materials and methods

**Fig. 1:**
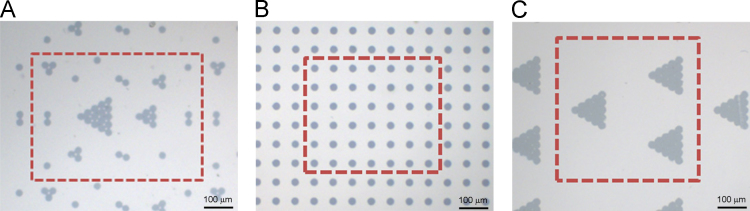
Bright-field optical micrographs of as-prepared micropatterns for the present cell studies. (A) Five types of microdomains with the numbers of microislands 1, 2, 3, 6 and 15 for collagen II immunofluorescent staining; (B) microdomains with single microislands for qRT-PCR detection of gene expression by single cells; (C) microdomains with the number of microislands 15 for qRT-PCR detection of gene expression by contacted cells.

The data provided here are bright-field micrographs of three sets of micropatterns with a similar microisland density (Fig. 1) and primer sequences of targeted genes for qRT-PCR (Table 1).

### 1.1 Micropatterns for cell studies

The method to prepare micropatterns had been reported previously by our group [2–11]. First, gold micropatterns were fabricated on glass with a pre-designed mask through photolithography. Then a bi-functional linker was grafted onto the gold with Au–S bond. After that, poly(ethylene glycol) diacrylate (PEGDA-700) mixed with a photo-initiator was coated on the glass surface. By UV irradiation, the macromonomer was cross-linked, and the gold micropatterns were transformed from the glass surface onto the PEG hydrogel by peeling-off. The micrograph presented in Fig. 1 was captured by a CCD in an inverted optical microscope (AXIOVERT 200, Zeiss).

### 1.2 Sequence of primers

The characteristic genes for the chondrogenic differentiation [12,13] were examined using qRT-PCR [14].

Original mRNA and genomic sequence was obtained from NCBI, then pasted into PerlPrimer [15]. After setting appropriate parameters (Primer temperature 58–62 °C, primer length 20–24 bases, amplicon size 100–300 bases), the most matched primers pairs were obtained. Then Primer-BLAST from NCBI [16] was used to check the specificity of the primers got from PerlPrimer. The sequences are listed in Table 1.

## Figures and Tables

**Table 1:** Sequences of forward (Fw) and reversed (Rv) primers designed in qRT-PCR for detection of mRNA expression of the genes of interest.

**Genes**	**Primer (5′-3′)**	
Collagen II	Fw	TGGAAGAGCGGAGACTACTG
	Rv	GTAGACGGAGGAAAGTCATCTGG
Collagen I	Fw	TCCTGCCGATGTCGCTATC
	Rv	CAAGTTCCGGTGTGACTCGTG
Aggrecan	Fw	TATGAGGATGGCTTCCACCAG
	Rv	AAGACCTCACCCTCCATCTC
SOX9	Fw	CTGAACGAGAGCGAGAAG
	Rv	TTCTTCACCGACTTCCTCC
HIF-1α	Fw	CTGAACGAGAGCGAGAAG
	Rv	TTCTTCACCGACTTCCTCC
GAPDH	Fw	GCTCTCTGCTCCTCCCTGTTCTAG
	Rv	TGGTAACCAGGCGTCCGAT
